# A global overview of renal registries: a systematic review

**DOI:** 10.1186/s12882-015-0028-2

**Published:** 2015-03-19

**Authors:** Frank Xiaoqing Liu, Peter Rutherford, Karen Smoyer-Tomic, Sarah Prichard, Suzanne Laplante

**Affiliations:** Baxter Healthcare Corporation, Deerfield, IL USA; Baxter Healthcare Corporation, Zürich, Switzerland; Oxford PharmaGenesis Inc, Newtown, PA USA

**Keywords:** Renal registry, Systematic review, Renal replacement therapy, End-stage renal disease, Hemodialysis, Peritoneal dialysis

## Abstract

**Background:**

Patient registries have great potential for providing data that describe disease burden, treatments, and outcomes; which can be used to improve patient care. Many renal registries exist, but a central repository of their scope, quality, and accessibility is lacking. The objective of this study was to identify and assess worldwide renal registries reporting on renal replacement therapy and compile a list of those most suitable for use by a broad range of researchers.

**Methods:**

Renal registries were identified through a systematic literature review and internet research. Inclusion criteria included information on dialysis use (yes/no), patient counts ≥300, and evidence of activity between June 2007 and June 2012. Public availability of information on dialysis modality, outcomes, and patient characteristics as well as accessibility of patient-level data for external research were evaluated.

**Results:**

Of 144 identified renal registries, 48 met inclusion criteria, 23 of which were from Europe. Public accessibility to annual reports, publications, or basic data was good for 17 registries and moderate for 22. Patient-level data were available to external researchers either directly or through application and review (which may include usage fees) for 13 of the 48 registries, and were inaccessible or accessibility was unknown for 25.

**Conclusions:**

The lack of available data, particularly in emerging economies, leaves information gaps about health care and outcomes for patients with renal disease. Effective multistakeholder collaborations could help to develop renal registries where they are absent, or enhance data collection and dissemination for currently existing registries to improve patient care.

**Electronic supplementary material:**

The online version of this article (doi:10.1186/s12882-015-0028-2) contains supplementary material, which is available to authorized users.

## Background

Patient registries provide an organized and standardized method to systematically collect observational data about specific groups of patients managed in routine clinical practice for a predetermined objective [1]. Registry data can help describe the natural history, epidemiology, and burden of a disease; and capture treatment site, regional, or national variations in treatment and outcomes to help evaluate safety, quality, and value of patient care [2]. Ultimately, data collected by patient registries may help researchers develop hypotheses about disease mechanisms or treatment approaches and inform health care policy thereby potentially improving quality. However, establishing a registry is a major undertaking, requiring sizeable resource investment from payers, health care providers (HCPs), and technical/administrative staff to initiate and maintain registry operation [1]. For countries with a limited pool of HCPs and resources, these obstacles may prohibit patient registry initiation.

End-stage renal disease (ESRD) and its current standard of care, renal replacement therapy (RRT; which includes dialysis and/or kidney transplantation) result in substantial economic and societal costs. Despite affecting up to 0.03% of the total population in developed countries, ESRD consumes up to 3% of annual healthcare budgets in many countries [3], and even more in the United States (US). In 2011, US Medicare-related total ESRD expenditures were $34.4 billion, accounting for 7.2% of the total Medicare budget while serving only 1.4% of Medicare patients. In the same year, the 1-year Medicare costs per patient using hemodialysis (HD) or peritoneal dialysis (PD) were approximately $88,000 and $72,000, respectively [4]. Global incidence rates for RRT range from 12 to 455 (median 130) per million population [5], with an increase of 6% to 7% annually, markedly outpacing the annual population growth rate of 1.2% [6]. The incidence of RRT varies considerably between countries due to factors like gross domestic product (GDP) per capita, percentage of GDP spent on health care, dialysis reimbursement rate, and the private for-profit share of dialysis provision [5]. These may contribute to faster growth rates in underdeveloped countries (in line with economic development) compared with wealthier countries where increases are driven more by ESRD incidence than access to RRT.

In the field of ESRD care, registries—some with long standing histories—have been widely established to collect data on patients undergoing RRT. Data from these registries can provide useful information to assess the effectiveness of treatment options, identify patients who could benefit from an existing or new treatment, and drive improvements in health care quality [7]. Moreover, registry data have revealed a variability in incidence, prevalence, and treatment modalities across the world [5], some of which may be explained by patient demand, and others by economic and provider-driven demand.

The global distribution, availability, and quality of renal registries are unclear, and the burden of ESRD in many low- and middle-income countries is not fully understood, due to a lack of national registries [8]. Therefore, there is a need to better understand the global distribution of ESRD registries, the type of data recorded and available, and the applicability of such data for improvements in clinical and policy decisions. An integrated, central repository of global renal registry data could help illustrate trends and outcomes from RRT, and ultimately lead to improved quality and consistency in care within a particular country, but also identify best practice across countries.

The objective of this research was to systematically identify and compile information about existing renal registries reporting RRT across the world, including an examination of their data elements and an evaluation of data accessibility. Accessibility was defined as availability of aggregate data, publication of periodic reports and/or literature in peer-reviewed journals, and accessibility to external researchers of anonymized patient-level data to investigate parameters like epidemiology, treatment patterns, and health outcomes for patients with ESRD. Additional objectives were as follows: to provide a resource that describes high-quality, accessible renal registries; identify regions with a need to commence or improve data collection; and increase availability of information to educate the public or inform clinical research by external investigators.

## Methods

Registries of patients with ESRD receiving dialysis were initially identified through a systematic literature review using Cochrane Renal Group standard search strings [9] to identify publications on renal registries through publicly available resources such as Medline/PubMed, Embase, the Cochrane Library, and the Centre for Reviews and Dissemination databases (Additional file 1). All human studies identified were included without date or language restrictions. Internet searches for renal registry websites were also performed. If the obtained information was in a language other than English, translation software (Google Chrome web browser, Google Translate, or Babylon free web-based translation services) or consultation with native language speakers were utilized for translation to English. Because this study did not involve the collection, use, or transmittal of individually identifiable data, Institutional Review Board (IRB) review or approval was not required. No formal review protocol exists for this study.

Based on the initial findings, internet research was performed to acquire publically available reports, publications, and supporting resources to apply inclusion criteria and identify registries suitable for detailed analysis. Observational studies similar in content to registries and meeting inclusion criteria were also considered. Inclusion criteria were registry activity for the 5-year period spanning June 2007 to June 2012, data collection for ≥300 patients, and availability of information on dialysis use and type. Thus, transplant-only registries were excluded from this study. We also excluded registries if their data were duplicated elsewhere, favoring the registry with the most recent or comprehensive data. An exception to this was the European Renal Association - European Dialysis and Transplant Association (ERA-EDTA) registry, which collates data from registries of over 30 countries. This registry was reported in aggregate; however, participating individual registries were analyzed separately if they contained more complete information than the ERA-EDTA itself.

Registries meeting inclusion criteria were further analyzed to obtain data on a comprehensive list of parameters as outlined in Table 1. Key variables included registry history; population; current activity; incidence, prevalence, and availability of aggregated or individual patient data; treatment characteristics (dialysis type and modality and transplant status); and clinical and economic outcomes. Publically available information rarely provided sufficient information about the scope of registry data elements collected or patient-level data availability for external researchers. Therefore, if the publically available information was insufficient, as part of the data collection process, up to 3 attempts were made to establish contact with registry personnel by email. An email template covering the same basic elements but tailored to each registry type, if needed, was used. Telephone contact was made if feasible for the all parties. For any registries that did not reply within 3 email attempts (using local language content as appropriate, translated as described above), further contact attempts were not made.

**Table 1:** **Data elements sought**

**Category**	**Data elements**
Basic registry information	Region, acronym, full name, website, country, year established, year of latest annual report, address, telephone/fax numbers, email, Chairperson, Director, history, current status (active or inactive), patient group, inclusion of pediatric data, geographical reach, use of quality control measures, inclusion of incident and/or prevalent patients, use of special inclusion sampling, inclusion of transplant and/or dialysis patients
Aggregate data	Availability, how to access, who can access, cost to access
Individual patient data	Availability, how to access, who can access, cost to access
Data source	Sector (public or private), RRT service provider, method of patient recruitment, means by which patients exit registry
Patient characteristics	Age, gender, race, ethnicity, body mass index, duration of ESRD, level of education, employment status, insurance
Comorbidities	Whether reported, specific comorbidities assessed
Treatment characteristics	Modality and submodality, dialysis-product information, dose of dialysis, vascular access method, if RRT initiation was unplanned or planned, if start date reported, length of time on dialysis, treatment costs, funding source
Outcomes	Laboratory results, quality of life (scale/instrument used), peritonitis rate, infection rate, other adverse events reported, survival data/all-cause mortality rates, renal failure-related mortality rates, any additional outcomes collected
ESRD, end-stage renal disease; RRT, renal replacement therapy	

A tailored assessment tool was developed to suit our purposes, ie, evaluating how renal registries can be used to steer public policies and improve patient care. The main focus of the assessment was on the availability of registry information to the general public and accessibility of patient-level data to external researchers. Specific registry features included amenability to collaboration with outside clinicians, payers, or researchers (academic or corporate) to enable use of registry data to address a variety of questions. General data accessibility was determined through both web-based search results and direct communications with registry personnel, and assigned to 1 of 3 categories as described below. All registries were assessed by at least 2 data analysts, with any noted discrepancies discussed and resolved.

**Accessibility** was defined as *good* (information including annual reports, publications, and aggregate data accessible via website, publicly available materials, or with assistance from registry staff); *moderate* (information in local language only or limited publicly available information including on website; with additional searches, basic information may be available in reports or in published research; more information may be accessible via third party collaborators); or *limited/unclear* (little or no information publicly available).

**Patient-level data** accessibility was determined in a similar manner, and categorized as *available* (to external researchers directly or through application and review, may include usage fees); *conditionally available* (eg, via third party collaborators); or *not available/unclear*.

**Treatment data** were ranked as *good* if dialysis sub-modality was available, *moderate* if modality but not submodality was available, and *limited* if the modality was not available or unclear.

**Outcome data** was categorized as *good* if mortality, survival and/or hospitalization, or complications data were available; *moderate* if mortality/survival or adverse events were not reported but surrogates such as laboratory result data were available; and *limited* if outcomes or surrogate data were not reported or unclear.

## Results

**Figure 1:**
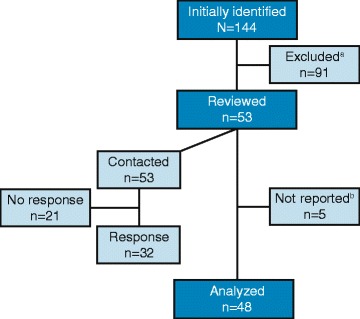
**Registry data analysis and reporting process.** ^a^A registry was excluded if it had been defunct for more than 5 years, had ≤300 participants, did not include data related to dialysis, was contained in and/or was redundant to another registry that was more appropriate for analysis. ^b^Reviewed databases not reported due to lack of available information (ie, insufficient data available through public sources and did not respond to queries for more information) included Israeli Society of Nephrology and Hypertension (The Official Israeli Nephrology Site), Madrid Registry of Renal Patients (although it did provide aggregate data to the Spanish Registry of Renal Patients), and Polish Registry of Renal Replacement Therapy in Children. The Scientific Registry of Transplant Recipients and Japan Renal Transplantation Registry were also excluded for focusing exclusively on transplant patients and providing no information on dialysis modalities.

The initial literature search yielded 1980 references that included data elements suggestive of renal registry activity (comprehensive search results are provided in Additional file 1). Screening titles and abstracts for renal registry names, combined with manual web searches, yielded 144 registries considered for further review (Figure 1). Subsequent application of the inclusion criteria resulted in 53 registries eligible for screening and review, all of which were contacted by email or telephone. In addition to the 91 registries immediately excluded for not meeting inclusion criteria, 3 registries (Israeli Society of Nephrology and Hypertension, Madrid Registry of Renal Patients, and Polish Registry of Renal Replacement Therapy in Children) were excluded due to insufficient available information, and 2 (Scientific Registry of Transplant Recipients and Japan Renal Transplantation Registry) were excluded due to lack of dialysis data, leaving 48 registries for detailed analysis. A list of available websites, representative publications, and reports for these 48 analyzed registries is provided in Additional file 2.

**Figure 2:**
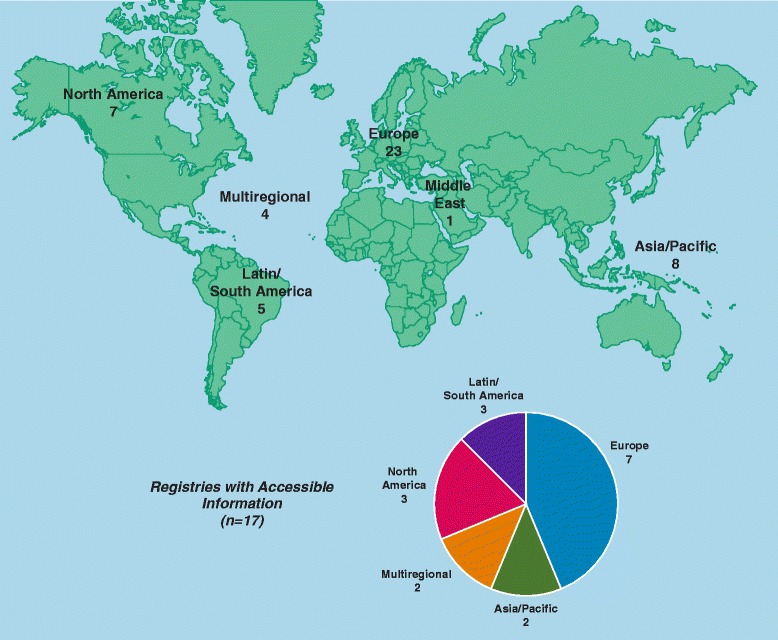
**Geographical distribution of analyzed registries (n = 48).**

The geographic distribution of the analyzed registries is shown in Figure 2. The majority were in Europe (23 [47.9%]), followed by the Asia/Pacific region (8 [16.7%]) and North America (7 [14.6%]). We also analyzed 4 registries (8.3%) that spanned multiple regions. At the time of this investigation no renal registries meeting inclusion criteria were identified in some Asian countries with large populations including India, the Philippines, and Indonesia. Moreover, no registries were identified in Africa at the time of this evaluation (see *Discussion*), although Morocco and Tunisia were included in the French Language Peritoneal Dialysis Registry.

Details of the 48 analyzed registries are summarized in Table 2. Overall, there was significant heterogeneity between organizational structures and histories between registries. Initiation dates ranged over 45 years (1963 to 2010), and registry history included *de novo* establishment as well as transition from or acquisition of preexisting registries by newer or larger registries. Few of the 48 analyzed registries provided information on patient racial origin/ethnicity, education, employment, insurance, dialysis access, dialysis start (planned vs unplanned), complications, quality of life (QoL), and treatment costs (details not shown).

**Table 2:** **Data summary for reported registries (n = 48)**

**Region**	**Registry Name (Common Abbreviation), Year Established**	**Accessibility**	**Patient-level data availability**	**Treatments**	**Outcomes**
Asia/Pacific (n = 8)	Australia and New Zealand Dialysis and Transplant Registry (ANZDATA), 1963	+++	++	+++	+++
	Hong Kong Renal Registry (HKRR), 1995	+	+	+++	+++
	Korean Renal Registry, 1985	++	++	+++	+++
	Malaysian National Renal Registry (NRR), 1993	++	+++	+++	+++
	Shanghai Dialysis Registry, 1996	+	+	++	+++
	Singapore Renal Registry, 2001	+++	++	+++	+++
	Taiwan Renal Registry Data System (TWRDS), 1987	+	+	+++	+
	Thailand Renal Replacement Therapy Registry (TRT), 1997	++	+	+++	+
Europe (n = 23)	Austrian Dialysis and Transplant Registry (OEDTR)*, ≤ 1990	++	+	++	+++
	Belgian Society of Nephrology (Dutch-speaking) (NBVN)*, ≤ 1996	++	++	+++	+
	Catalan Renal Registry (RMRC)*, 1984	++	+	++	+++
	Danish Registry on Regular Dialysis and Transplantation (DNSL)*, 1990	+++	+++	+++	+++
	Dutch Renal Registry (RENINE), 1986	++	+	++	+++
	European Renal Association - European Dialysis and Transplant Association (ERA-EDTA), 1963	+++	++	+++	+++
	Finnish Registry for Kidney Diseases*, 1964	+++	+++	+++	+++
	French Renal Epidemiology and Information Network (REIN), 2002	++	+	+++	+++
	Greek Registry (Hellenic Society of Nephrology)*, 2000	+	+	+	+
	Groupement des Nephrologues Francophones de Belgique (GNFB), 1995	++	+	++	+++
	Italian Dialysis and Transplant Registry (RIDT)*, 1996	+++	+	++	+++
	Norwegian Renal Registry*, 1994	++	+++	++	+++
	Peritoneal Dialysis Board Registry (GSDP), ≤ 2001	++	+	+++	+++
	Portuguese Society of Nephrology*, 1997	++	+	++	+++
	Romanian Renal Registry (RRR)*, 1993	+	++	++	+
	Russian Registry*, 1998	++	+	+++	+++
	Scottish Renal Registry (SRR)*, 1991	+++	+++	+++	+++
	Spanish Society of Nephrology Register (Peritoneal Dialysis Registry) (SEN), 1997	++	+	++	++
	Spanish Society of Nephrology Register (Records of Renal Patients [GRER]), 1997	++	+	+++	+++
	Swedish Renal Registry (SNR/SRR)*, 2007	++	++	++	+++
	Turkish National Registry (TSNRR)*, 1990	++	+	+++	+++
	United Kingdom Renal Registry (UKRR)*, 1997	+++	+++	+++	+++
	Valencian Renal Registry*, 1992	+++	++	+++	+++
Latin/South America (n = 5)	Sociedad Argentina de Nefrologia (SAN)/Instituto Nacional Central Unico Coordinator de Ablación e Implante (INCUCAI); 2004	+++	+++	+++	+++
	Brazilian Registry of Dialysis (RBD/SBN), 1998	+	+	++	+++
	Colombia Healthcare Database, ≤ 2008	+++	+++	++	+
	Latin American Dialysis and Transplantation Registry (SLANH/RLDT), 1991	++	+	++	+
	Uruguayan Registry of Dialysis, 1981	+++	+++	+++	+++
Middle East (n = 1)	United Arab Emirates Renal Diseases Registry, 1980	+	+	+	+
North America (n = 7)	British Columbia Renal Database - Patient Records and Outcome Management Information System (PROMIS), 2003	+	+++	++	+
	Canadian Organ Replacement Register (CORR), 1994	+++	+++	+++	++
	Canadian Pediatric End-Stage Renal Disease Database, ≤ 2010	++	+	+	+
	Database of the Renal Research Institute (MONDO), 2000	++	+	++	+
	North American Pediatric Renal Trials and Collaborative Studies (NAPRTCS), 1987 transplant only, HD/PD as of 1992	+++	+	+++	+++
	The Renal Disease Registry (TRDR/ORN), 1981	++	+	+++	+++
	US Renal Data System (USRDS), 1988	+++	++	+++	+++
Multiregional (n = 4)	Dialysis Outcomes and Practice Patterns Study (DOPPS), 1996	+++	++	++	++
	French Language Peritoneal Dialysis Registry (RDPLF), ≤ 1995	+++	+++	+++	+++
	International Pediatric Peritoneal Dialysis Network Registry (IPPN), 2007	+	+++	+++	+
	International Quotidian Dialysis Registry (IQDR), 2003	++	+	+++	+

*Registry contributes to ERA-EDTA.

Full references to online registry resources are provided in Additional file 2.

**Accessibility**: +++(Good): Information including annual reports, publications, and aggregate data accessible via website, publicly available records, or with assistance from registry staff; ++(Moderate): Information in local language only or limited publicly available information including on website; with additional searches, basic information may be available in reports or in published research; more information may be accessible via third party collaborators (eg, registry researchers or local academics); +(Limited/unclear): very limited information available publicly or unclear.

**Patient-level data**: +++Available to external researchers directly or through application and review, may include usage fee; ++Conditional access, eg, via third party collaborators; +Not available to external researchers or access process unclear.

**Treatment**: +++ Submodality available; ++Modality available but not submodality; +Modality not available or availability unclear.

**Outcome data**: +++ Mortality/survival and/or hospitalization/complications data available; ++ Mortality/survival or hospitalization/complications not reported; surrogates such as laboratory result data reported; +No reported outcomes or surrogate data or availability unclear.

Among the characteristics assessed for the 48 registries, the focus was on 4 major areas: public availability of registry data such as annual reports or aggregate data, availability of individual patient-level data to outside researchers, RRT modality and submodality information, and details on reported patient outcomes with a focus on mortality. As shown in Table 2, we categorized these 4 parameters using a low-medium-high (+/++/+++) rating scheme as described in the *Methods*.

Overall, 17 registries (35.4%) had good public accessibility and 22 registries had moderate accessibility (48.5%) to general information and/or aggregate data. Additional details about registries considered to have good public accessibility to information are provided in Table 3, with their regional distributions depicted graphically in a pie chart in Figure 2. Patient- level data were not accessible to external researchers (or access was unclear) for the majority of registries (25 of 48, [52.1%]). Patient-level data were available to external researchers either directly or through application and review (which may include usage fees) for 13 registries (27.1%), and 10 (20.8%) had conditional data availability. Examples of conditional data availability include the Korean Renal Registry (available for academic purposes only), the US Renal Data System (USRDS; available through a registry coordinating center or through a third party approved research team and request), and the Dialysis Outcomes and Practice Patterns Study (DOPPS; potentially available to corporate sponsors). Latin/South America had the highest proportion of registries with patient-level data accessible to external researchers (3 of 5 [60.0%]), followed by multiregional registries (2 of 4 [50%]), North America (2 of 7 [28.6%), Europe (5 of 23 [21.7%]), and Asia (1 of 8 [12.5%]). No patient-level data were available in the Middle East. We identified 7 registries allowing external access to patient-level data that reported RRT modality and submodality and had comprehensive outcomes data. These were Danish Registry on Regular Dialysis and Transplantation, Finnish Registry for Kidney Diseases, Scottish Renal Registry, French Language Peritoneal Dialysis Registry, United Kingdom Renal Registry, Argentina Society of Nephrology/Central National Institute Unique Coordinator of Ablation and Implant, and the Uruguayan Registry of Dialysis.

**Table 3:** **Renal replacement modality for registries with good accessibility to general information** ^**1**^ (**n = 17**)

**Full name**	**Country**	**Year established**	**Patient-level data available?**	**Renal replacement modality reported** ^**2**^		
				**HD**	**PD**	**Transplant**
Australia and New Zealand Dialysis and Transplant Registry	Australia, New Zealand	1963	Yes^3^	Home, Clinic	CAPD, APD	Yes
Canadian Organ Replacement Register	Canada	1994	Yes^3^	Home, Clinic	CAPD, APD	Yes
Colombia Healthcare Database	Colombia	2008^4^	Yes	Unspecified	Unspecified	Yes
Danish Registry on Regular Dialysis and Transplantation	Denmark	1990	Yes^3^	Home, Clinic	APD	Yes
Dialysis Outcomes and Practice Patterns Study (DOPPS), 1996	Australia, Belgium, Canada, France, Germany, Japan, Italy, New Zealand, Spain, Sweden, UK, USA	1996	Conditional^5^	Clinic	Unspecified	No
European Renal Association - European Dialysis and Transplant Association (ERA-EDTA),	Europe	1963	Conditional^5^	Home, Clinic^6^	CAPD, APD	Yes
Finnish Registry for Kidney Diseases	Finland	1964	Yes^3^	Home, Clinic	CAPD, APD	Yes
French Language Peritoneal Dialysis Registry (RDPLF)	Belgium, France, Switzerland, Morocco, Tunisia	≤1995	Yes^3^	Unspecified	CAPD, APD	Yes
Italian Dialysis and Transplant Registry	Italy	1996	No	Clinic	CAPD, APD	Yes
North American Pediatric Renal Trials and Collaborative Studies (NAPRTCS),	USA primarily, Canada, Latin America (countries not specified)	1987 transplant only, HD/PD as of 1992	Unclear	Clinic	CAPD/APD/IPD	Yes
Scottish Renal Registry	Scotland	1991	Yes^3^	Home, Clinic	CAPD, APD	Yes
Singapore Renal Registry	Singapore	2001	Yes^3^	Clinic	CAPD, APD	Yes
Sociedad Argentina de Nefrologia (SAN)/Instituto Nacional Central Unico Coordinator de Ablación e Implante (INCUCAI); 2004	Argentina	2004	Yes	Unspecified	CAPD/APD	Yes
United Kingdom Renal Registry	UK	1997	Yes^3^	Home, Clinic	CAPD, APD	Yes
Uruguayan Registry of Dialysis	Uruguay	1981	Yes	Clinic	CAPD, APD	Yes
US Renal Data System	USA	1988	Conditional^5^	Home, Clinic	CAPD, APD	Yes
Valencian Renal Registry	Italy	1992	Unclear	Clinic	CAPD, APD	Yes

APD, automated peritoneal dialysis; CAPD, continuous ambulatory peritoneal dialysis; HD, hemodialysis; PD, peritoneal dialysis.

^1^Data derived from publicly accessible information and may not be comprehensive or reflect full range of collected information.

^2^Hemofilration use not available except for Finnish Registry for Kidney Diseases and Italy (12 of 20 regions as per ERA-EDTA). Dialysis product information not available for any registry listed.

^3^Application/inquiry process required.

^4^Year of are earliest publicly available data. Year of registry establishment unclear.

^5^Access conditional via registry staff led research or collaborative partner.

^6^Large majority of data are in-center/clinic dialysis.

Treatment data, including general categories PD and HD, were available from most registries. However, the number reporting submodalities of PD (continuous ambulatory PD [CAPD], automatic PD [APD]); type of HD (clinic versus home; high-dose HD); or hemofiltration and hemodiafiltration was more limited. When assessing RRT data availability, submodality details were identified for more than half (28 [58.3%]) of all registries, whereas 17 (35.4%) provided modality but not submodality data. For the remaining 3 registries (6.3%), modality data was unavailable or unclear. Treatment cost data (not shown) was identified for only 4 (8.3%) analyzed registries: USRDS, Thailand Renal Replacement Therapy Registry, Colombia Healthcare Database, and Spanish Society of Nephrology Register (Records of Renal Patients).

Outcome data were categorized in terms of availability of mortality, survival, hospitalization, or complications data. Registries that provided data on at least 1 of these outcomes were rated as having the best outcomes data; whereas, the next tier included registries offering partial information and/or surrogate outcome data like laboratory results. Over half (32 [66.7%]) of the registries provided mortality, hospital or complications outcomes data; the largest proportion of which came from Europe (19 of 23 [82.6%]), followed by Asia-Pacific (6 of 8 [75.0%]), Latin/South America (3 of 5 [60.0%]), North America (3 of 7 [42.9%]), and none in the Middle East. The multiregion French Language Peritoneal Dialysis Registry had good availability of outcomes as well as treatment data with most centers in France but also from other French speaking countries in Europe and North Africa.

## Discussion

Based on our survey of renal registries, we identified 144 for further evaluation, and analyzed 53 in detail. Subsequently, 3 were excluded for not meeting inclusion criteria and 2 more for lack of available information, resulting in 48 registries analyzed and reported. Most of the 48 registries provided information on transplantation and dialysis modality, yet very few provided treatment cost data or clinical or patient outcomes other than on mortality. Of the 48 analyzed registries, 17 were deemed to provide good public access to information such as detailed reports and publications, and 23 were determined to allow access to individual-level patient data as required to conduct real-world research. Several of these registries offered data analysis services through registry statisticians as part of a contracted research project or via collaboration with registry researchers.

In the era of patient-centered care, registries with complete clinical, humanistic, and economic information on patients are essential to help monitor and improve patient outcomes. Health care systems should have a vested interest in supporting registry activity, particularly in acquiring, maintaining, and analyzing the data. High-quality registries provide valuable information that can impact health care procedures, policy, and ultimately, population health. A number of therapeutic areas provide powerful examples of how registry data can positively affect patient care. For example, the European Network of Cancer Registries publishes fact sheets and treatment recommendations, provides training courses, and holds international conferences to present the latest data and recommendations to improve patient assessment and care [10]. In the US, the National Cardiovascular Disease Registry provides evidence-based quality improvement solutions for HCPs and guidance on facility-based issues like accreditations, cost control, reimbursements, and securing and retaining high-level clinicians [11]. Similar programs could be of significant benefit for ESRD patients, some of which have already begun. For example, cost data ascertained through the USRDS helped drive a recent shift in health care policy with the End-Stage Renal Disease Prospective Payment System, designed to better manage costs for ESRD treatment [12]. Data from the ESRD Networks in the United States (www.esrdncc.org) support the Fistula First Breakthrough Initiative, introduced to advance arteriovenous fistula placement and reduce central venous catheter use to improve patient survival and QoL by providing a number of incentives for both patients and health care providers (see http://www.fistulafirst.org). The peritoneal dialysis first initiative by the Universal Health Coverage Scheme (UCS) in Thailand [13] and the call to action by nephrologists from Australia and New Zealand use ESRD registry data to address observed low survival rates with PD [14]. Finally, the Latin American Dialysis and Renal Transplant Registry along with the Latin American Society of Nephrology and Hypertension have supported a sustained effort within the entire Latin American nephrology community by hosting seminars for the creation and/or improvement renal registries throughout Latin America [15,16].

We identified a number of gaps in renal registry coverage, revealing that, despite large ESRD patient populations and an established framework for data collection in many countries, opportunities for tracking patient care are being missed. First, although many renal registries have been established around the world, less than half that met our analysis criteria provided information accessible to the general public, and few had suitable patient-level data availability and access needed to generate evidence to support improved patient care. Data on the burden of dialysis on patients and caregivers and on societal and payer costs of care were also limited. Second, few registries were identified in areas with emerging economies; the majority being based in high-income countries, creating geographic gaps in coverage. In much of the Asia-Pacific region, the Middle East, and Africa, registries were either absent entirely or had limited data or poor accessibility for outside research. Additionally, some of the world’s most populous health care systems like China, Russia, and India currently have limited (eg, limited to 1 city like Moscow or Shanghai) or inadequate registries. Of particular note is India, which has an age-adjusted incidence of ESRD of 232 per million population [17]. Although some ESRD patients are captured in the India chronic kidney disease registry, [18] there is no specific registry for ESRD.

We also identified a lack of uniformity in the type and quality of data collected and in how modalities were reported. For example, some registries reported high-level information on HD and PD submodalities, but did not provide details on associated outcomes (eg, the USRDS reported the percentage distribution of CAPD vs. APD, but not outcomes from these). Limited data are available in registries to compare the use of, or outcomes in, patients receiving hemodialysis vs. hemodiafiltration in Europe despite its wide availability across that region. Thus, the performance of different dialysis methods in a real-world clinical setting is poorly understood due to limitations in existing registry data. This is a particular concern considering that registries are generally not organized or funded to integrate new data elements [1], and thus information on submodalities may be missed as dialysis therapies evolve. Current treatment data need to be incorporated into registries to address this gap in dialysis treatment information.

Finally, we also encountered a general difficulty in accessing detailed registry data; both aggregated data for the general public, and patient-level data for external research. We concluded that research using many of the identified renal registries with high-quality information would be restricted by both limited data collection and dissemination as well as access to external researchers. Even for registries that might be receptive to collaboration, registry personnel may lack time or resources to conduct and publish studies, resulting in a backlog of information that is not disseminated. Although there initially appeared to be a large number (144) of renal registries globally, only a small minority had comprehensive patient-level data readily accessible for external research, and these tended to be in high-income countries. These restrictions on registry data accessibility limit opportunities for real-world research of treatment patterns and associated outcomes, potentially missing opportunities to improve patient care.

This study has several limitations. First, the analysis was completed as of June 2012, and thus some newer registries like the recently established South African Renal Registry (first annual report published in 2014) were not included. It is also possible that the registries we examined here added or made available new information since our analysis was completed. Second, if a registry was operating with no public-facing information like a website, periodic reports, or literature publications, it could have gone undetected. Finally, it should be noted that the ranking system (eg, as shown in Table 2) was inherently subjective, based on best information available at the time and the researchers’ interpretation of this information.

Based on the study findings, a number of recommendations emerged. Gaps in registry coverage, data collection and completeness of case ascertainment, and accessibility present an opportunity for more productive collaborations to collect relevant data, implement quality and standardization procedures, and provide broad access to comprehensive aggregate information and anonymized patient-level data to facilitate the advancement of patient care. Communication with local governments and health authorities can be initiated to begin to address data gaps identified in this analysis to improve data quality and accessibility. New data elements and key patient information (eg, detailed patient information; dialysis access, new modalities, and initiation dates; QoL; and costs) could be incorporated into existing registries. Additionally, specific mechanisms to report and improve data quality, resources to maintain and update registries, and increased accessibility are needed. For example, inroads to existing, seemingly difficult-to-access renal registries could be developed to expand opportunities for more thorough data analysis and outcomes research, thus leveraging the existing registry infrastructure while building collaboration. Through multistakeholder collaboration, opportunities could be pursued to develop or expand renal registries in large but poorly represented countries such as India, China, and the Russian Federation. These partnerships could help address the human resource and financial issues inherent in maintaining a high-quality registry. Additionally, established registries or societies like the International Society of Nephrology could potentially assist with the formation of new registries in less developed countries by providing assistance with process and protocol development, information technology expertise, and infrastructure required to initiate patient registries. Finally, global guidelines for uniform and centralized data collection and quality assurance across all geographic areas could be proposed. Current examples of this type of initiative include the recently established EURODOPPS (a partnership between the ERA-EDTA and Dialysis Outcomes and Practice Patterns) [19,20], the Registry of Patient Registries by the US Agency for Healthcare Research and Quality; and the ERA-EDTA/SLANH Registries Fellowships [21]. These programs help expand knowledge on the epidemiology of ESRD and treatment approaches by leveraging strengths and pooling the resources of multiple initiatives. This can help address scientific and policy questions, facilitate research, compare and pool results between registries, and use the strengths contained in multiple registries to generate new insights into treatments for ESRD patients.

## Conclusions

Existing registries can provide valuable information about ESRD patients and inform or help monitor the implementation of policy for renal care, but registries vary in terms of types of data collected, data quality, and accessibility. Insufficient information is available on the range of new dialysis modalities, as well as on socioeconomic factors, comorbidities, QoL and clinical outcomes, and costs of care. Even when these data are collected, dissemination to the public may be poor, and data accessibility is often difficult for external researchers. Limited data are available from emerging economies, resulting in information gaps about health care and outcomes for large populations of ESRD patients. Effective collaborations or partnerships with existing registries or societies could help to develop renal registries where they currently do not exist, or enhance data collection and dissemination for currently existing registries.

## Additional files

Additional file 1:
**Renal Registries Comprehensive Search Results.**
Additional file 2:
**Additional Resources for Analyzed Renal Registries.**

## Abbreviations

APD: Automated PD
CAPD: Continuous ambulatory PD
DOPPS: Dialysis Outcomes and Practice Patterns Study
ERA-EDTA: European Renal Association - European Dialysis and Transplant Association
ESRD: End-stage renal disease
GDP: Gross domestic product
HCP: Health care professional
HD: Hemodialysis
PD: Peritoneal dialysis
QoL: Quality of life
RRT: Renal replacement therapy
US: United States
USRDS: The United States Renal Data System
