# Supplementary material for: A global overview of renal registries: a systematic review
Source: BMC Nephrol. 2015 Mar 19;16:31. doi: 10.1186/s12882-015-0028-2 (PMC4377012; doi:10.1186/s12882-015-0028-2)
Supplement: Additional file 1: — Renal Registries Comprehensive Search Results. [file 12882_2015_28_MOESM1_ESM.pdf]

Appendix 1. Renal Registries Comprehensive Search Results

All Databases Combined

PubMed	EMBASE	Cochrane	CRD	Combined, duplicates removed
1206	1837	20	3	1980

Individual Database Searches

PubMed

Search (Note: all searches restricted to 'Humans'; no language restriction)		Hits
	**Renal terms**	
#1	hematuria	16,280
#2	hemoglobinuria	3412
#3	kidney diseases	332,022
#4	proteinuria	32,937
#5	renal[Text Word] OR kidney*[Text Word]	517,598
#6	hemodialysis[Text Word]	40,339
#7	hemofiltrat*[Text Word]	4670
#8	CAPD[Text Word] OR CCPD[Text Word] OR APD[Text Word]	7111
#9	dialysis[Text Word]	101,086
#10	glomeruloscler*[Text Word]	5556
#11	glomeruloneph*[Text Word]	29,362
#12	nephritis[Text Word] OR nephrotic[Text Word] OR nephrosis[Text Word] OR nephropathy*[Text Word]	55,112
#13	proteinuri*[Text Word]	25,555
#14	#1 OR #2 OR #3 OR #4 OR #5 OR #6 OR #7 OR #8 OR #9 OR #10 OR #11 OR #12 OR #13	607,173
	**Registry terms**	
#15	registry[Title] OR registries[Title]	9423
#16	database [Title]	6799
#17	#15 OR #16	16,136
	**Combination**	
#18	#14 AND #17 NOT editorial[Publication Type]	1206

EMBASE

**Search** (Note: all searches restricted to 'Humans'; no language restriction)		**Hits**
	**Renal terms**	
#1	hematuria	24,299
#2	hemoglobinuria	3921
#3	'kidney disease'/exp	443,557
#4	proteinuria	47,129
#5	renal OR kidney*	751,494
#6	hemodialysis OR haemodialysis	75,224
#7	hemofiltrat* OR haemofiltrat*	6469
#8	CAPD OR CCPD OR APD	8488
#9	dialysis	95,683
#10	glomeruloscler*	7596
#11	glomeruloneph*	31,634
#12	nephritis OR nephrotic OR nephrosis OR nephropathy*	115,941
#13	proteinuri*	47,451
#14	#1 OR #2 OR #3 OR #4 OR #5 OR #6 OR #7 OR #8 OR #9 OR #10 OR #11 OR #12 OR #13	792,707
	**Registry terms**	
#15	registry:ti OR register:ti OR registries:ti	13,813
#16	database:ti OR 'data base':ti	7711
#17	#15 OR #16	21,380
	**Combination**	
#18	#14 AND #17 NOT editorial:it	1837

Cochrane Library

**Search**		**Hits**
	**Renal terms**	
#1	hematuria	465
#2	hemoglobinuria	54
#3	MeSH descriptor **Kidney Diseases** explode all trees	8387
#4	proteinuria	1975
#5	renal OR kidney*	34,428
#6	hemodialysis OR haemodialysis	4772
#7	hemofiltrat* OR haemofiltrat*	558
#8	CAPD OR CCPD OR APD	745
#9	dialysis	8138
#10	glomeruloscler*	158
#11	glomeruloneph*	871
#12	nephritis OR nephrotic OR nephrosis OR nephropathy*	4290
#13	proteinuri*	2040
#14	#1 OR #2 OR #3 OR #4 OR #5 OR #6 OR #7 OR #8 OR #9 OR #10 OR #11 OR #12 OR #13	37,729
	**Registry terms**	
#15	(registry OR registries):ti	272
#16	(database):ti	344
#17	#15 OR #16	613
	**Combination**	
#18	#14 AND #17	20

Centre for Reviews and Dissemination

**Search**		**Hits**
	**Renal terms**	
#1	hematuria	26
#2	hemoglobinuria	8
#3	Kidney Diseases	278
#4	proteinuria	138
#5	renal OR kidney*	2275
#6	hemodialysis OR haemodialysis	321
#7	hemofiltrat* OR haemofiltrat*	38
#8	CAPD OR CCPD OR APD	30
#9	dialysis	637
#10	glomeruloscler*	3
#11	glomeruloneph*	56
#12	nephritis OR nephrotic OR nephrosis OR nephropathy*	258
#13	proteinuri*	142
#14	#1 OR #2 OR #3 OR #4 OR #5 OR #6 OR #7 OR #8 OR #9 OR #10 OR #11 OR #12 OR #13	2542
	**Registry terms**	
#15	(registry):TI OR (registries):TI	37
#16	(database):TI	40
#17	#15 OR #16	75
	**Combination**	
#18	#14 AND #17	3
