# Supplementary material for: A global overview of renal registries: a systematic review
Source: BMC Nephrol. 2015 Mar 19;16:31. doi: 10.1186/s12882-015-0028-2 (PMC4377012; doi:10.1186/s12882-015-0028-2)
Supplement: Additional file 2: — Additional Resources for Analyzed Renal Registries. [file 12882_2015_28_MOESM2_ESM.pdf]

Appendix 2: Additional Resources for Analyzed Renal Registries

<u>ASIA/PACIFIC</u>

Australia and New Zealand Dialysis and Transplant Registry (ANZDATA)
http://www.anzdata.org.au/v1/index.html

Foote, C., P. A. Clayton, D. W. Johnson, M. Jardine, P. Snelling, and A. Cass. "Impact of Estimated Gfr Reporting on Late Referral Rates and Practice Patterns for End-Stage Kidney Disease Patients: A Multilevel Logistic Regression Analysis Using the Australia and New Zealand Dialysis and Transplant Registry (Anzdata)." *Am J Kidney Dis* (Apr 29 2014).

McDonald, S. P., and G. R. Russ. "Australian Registries-Anzdata and Anzod." *Transplant Rev (Orlando)* 27, no. 2 (Apr 2013): 46-9.

Shahir, A. K., N. Briggs, J. Katsoulis, and V. Levidiotis. "An Observational Outcomes Study from 1966-2008, Examining Pregnancy and Neonatal Outcomes from Dialysed Women Using Data from the Anzdata Registry." *Nephrology (Carlton)* 18, no. 4 (Apr 2013): 276-84.

Hong Kong Renal Registry (HKRR)

Cheung, C. Y., M. F. Lam, K. M. Chow, W. Lee, Y. L. Cheng, S. K. Yuen, P. N. Wong, *et al.* "Hepatocellular Carcinoma after Kidney Transplantation: Analysis of Hong Kong Renal Registry." *Ren Fail* 36, no. 6 (Jul 2014): 865-9.

Cheung, C. Y., M. F. Lam, K. H. Chu, K. M. Chow, K. Y. Tsang, S. K. Yuen, P. N. Wong, *et al.* "Malignancies after Kidney Transplantation: Hong Kong Renal Registry." *Am J Transplant* 12, no. 11 (Nov 2012): 3039-46.

Ho, Y. W., C. B. Leung, B. Y. Choy, K. S. Fung, P. N. Wong, Y. L. Cheng, W. M. Lai, *et al.* "Renal Registry and Peritoneal Dialysis Management: The Hong Kong Perspective." *Perit Dial Int* 28 Suppl 3 (Jun 2008): S12-4.

Korean Renal Registry
http://www.ksn.or.kr/english/esrd.php

Jin, D. C. "Current Status of Dialysis Therapy in Korea." *Korean J Intern Med* 26, no. 2 (Jun 2011): 123-31.

Lee, H. B. "History of Nephrology and Renal Replacement Therapy in Korea." *J Nephrol* 24 Suppl 17 (May-Jun 2011): S93-6.

Malaysian National Renal Registry (NRR)
http://www.msn.org.my/nrr/

Stewart, J. H., M. R. McCredie, S. M. Williams, S. S. Fenton, L. Trpeski, S. P. McDonald, K. J. Jager, *et al.* "The Enigma of Hypertensive Esrd: Observations on Incidence and Trends in 18 European, Canadian, and Asian-Pacific Populations, 1998 to 2002." *Am J Kidney Dis* 48, no. 2 (Aug 2006): 183-91.

Shanghai Dialysis Registry
http://sh.cnrds.org/

Yao, Q., W. Zhang, and J. Qian. "Dialysis Status in China: A Report from the Shanghai Dialysis Registry (2000-2005)." *Ethn Dis* 19, no. 1 Suppl 1 (Spring 2009): S1-23-6.

Yao, Q., W. Zhang, and J. Qian. "Peritoneal Dialysis in Shanghai." *Perit Dial Int* 28 Suppl 3 (Jun 2008): S42-5.

Singapore Renal Registry
http://www.nrdo.gov.sg/page.aspx?id=370

Jin, A., W. P. Koh, K. Y. Chow, J. M. Yuan, and T. H. Jafar. "Smoking and Risk of Kidney Failure in the Singapore Chinese Health Study." *PLoS One* 8, no. 5 (2013): e62962.

Vathsala, A., and K. Y. Chow. "Renal Transplantation in Singapore." *Ann Acad Med Singapore* 38, no. 4 (Apr 2009): 291-9.

Taiwan Renal Registry Data System (TWRDS)

Chou, C. Y., C. C. Liang, H. L. Kuo, C. T. Chang, J. H. Liu, H. H. Lin, I. K. Wang, Y. F. Yang, and C. C. Huang. "Comparing Risk of New Onset Diabetes Mellitus in Chronic Kidney Disease Patients Receiving Peritoneal Dialysis and Hemodialysis Using Propensity Score Matching." *PLoS One* 9, no. 2 (2014): e87891.

Huang, C. C., K. F. Cheng, and H. D. Wu. "Survival Analysis: Comparing Peritoneal Dialysis and Hemodialysis in Taiwan." *Perit Dial Int* 28 Suppl 3 (Jun 2008): S15-20.

Lin, H. H., C. W. Tsai, P. H. Lin, K. F. Cheng, H. D. Wu, I. K. Wang, C. Y. Lin, W. Chen, and C. C. Huang. "Survival Analysis of Pediatric Dialysis Patients in Taiwan." *Nephrology (Carlton)* 17, no. 7 (Sep 2012): 621-7.

Thailand Renal Replacement Therapy Registry (TRT)
http://thairrtregistry.org/

Krairittichai, U., T. Supaporn, P. Aimpun, A. Wangsiripaisan, A. Chaiprasert, A. Sakulsaengprapha, A. Chittinandana*, et al.* "Anemia and Survival in Thai Hemodialysis Patients: Evidence from National Registry Data." *J Med Assoc Thai* 89 Suppl 2 (Aug 2006): S242-7.

Teerawattananon, Y., M. Mugford, and V. Tangcharoensathien. "Economic Evaluation of Palliative Management Versus Peritoneal Dialysis and Hemodialysis for End-Stage Renal Disease: Evidence for Coverage Decisions in Thailand." *Value Health* 10, no. 1 (Jan-Feb 2007): 61-72.

Thanachartwet, V., W. Phumratanaprapin, V. Desakorn, D. Sahassananda, Y. Wattanagoon, A. Chaiprasert, P. Aimpun, and T. Supaporn. "Viral Hepatitis Infections among Dialysis Patients: Thailand Registry Report." *Nephrology (Carlton)* 12, no. 4 (Aug 2007): 399-405.

EUROPE

Austrian Dialysis and Transplant Registry
http://www.nephro.at/JB_all.htm

Kainz, A., G. Goliasch, F. Wiesbauer, T. Binder, G. Maurer, H. J. Nesser, R. Mascherbauer, *et al.* "Left Atrial Diameter and Survival among Renal Allograft Recipients." *Clin J Am Soc Nephrol* 8, no. 12 (Dec 2013): 2100-5.

Belgian Society of Nephrology (Dutch-speaking) (NBVN)
http://www.nbvn.be/

Stewart, J. H., M. R. McCredie, S. M. Williams, S. S. Fenton, L. Trpeski, S. P. McDonald, K. J. Jager, *et al.* "The Enigma of Hypertensive Esrd: Observations on Incidence and Trends in 18 European, Canadian, and Asian-Pacific Populations, 1998 to 2002." *Am J Kidney Dis* 48, no. 2 (Aug 2006): 183-91.

Catalan Renal Registry (RMRC)
http://www20.gencat.cat/portal/site/canalsalut/menuitem.41e04b39494f1be3ba963bb4b0c0e1a0/?vgnextoid=6799a0aee474e210VgnVCM2000009b0c1e0aRCRD&vgnextchannel=6799a0aee474e210VgnVCM2000009b0c1e0aRCRD&vgnextfmt=default

Comas, J., E. Arcos, C. Castell, A. Cases, A. Martinez-Castelao, T. Donate, and E. Esmatjes. "Evolution of the Incidence of Chronic Kidney Disease Stage 5 Requiring Renal Replacement Therapy in the Diabetic Population of Catalonia." *Nephrol Dial Transplant* 28, no. 5 (May 2013): 1191-8.

Mauri, J. M., M. Cleries, and E. Vela. "Design and Validation of a Model to Predict Early Mortality in Haemodialysis Patients." *Nephrol Dial Transplant* 23, no. 5 (May 2008): 1690-6.

Soler, M. J., N. Montero, M. J. Pascual, C. Barrios, E. Marquez, M. A. Orfila, H. Cao, *et al.* "Age May Explain the Association of an Early Dialysis Initiation with Poor Survival." *QJM* (May 22 2014).

Danish Registry on Regular Dialysis and Transplantation (NRDT)
http://www.nephrology.dk/publikationer.htm

Hommel, K., S. Rasmussen, A. L. Kamper, and M. Madsen. "Regional and Social Inequalities in Chronic Renal Replacement Therapy in Denmark." *Nephrol Dial Transplant* 25, no. 8 (Aug 2010): 2624-32.

Hommel, K., S. Rasmussen, M. Madsen, and A. L. Kamper. "The Danish Registry on Regular Dialysis and Transplantation: Completeness and Validity of Incident Patient Registration." *Nephrol Dial Transplant* 25, no. 3 (Mar 2010): 947-51.

Kristensen, S. L., E. L. Fosbol, A. L. Kamper, L. Kober, K. Hommel, M. Lamberts, S. Z. Abildstrom, *et al.* "Use of Nonsteroidal Anti-Inflammatory Drugs Prior to Chronic Renal Replacement Therapy Initiation: A Nationwide Study." *Pharmacoepidemiol Drug Saf* 21, no. 4 (Apr 2012): 428-34.

Dutch Renal Registry (RENINE)
https://www.renine.nl/

Hofstra, J. M., and J. F. Wetzels. "Introduction of a Cyclophosphamide-Based Treatment Strategy and the Risk of Esrd in Patients with Idiopathic Membranous Nephropathy: A Nationwide Survey in the Netherlands." *Nephrol Dial Transplant* 23, no. 11 (Nov 2008): 3534-8.

Liem, Y. S., J. B. Wong, M. G. Hunink, F. T. de Charro, and W. C. Winkelmayer. "Comparison of Hemodialysis and Peritoneal Dialysis Survival in the Netherlands." *Kidney Int* 71, no. 2 (Jan 2007): 153-8.

Weijnen, T. J., H. W. van Hamersvelt, P. M. Just, D. G. Struijk, Y. I. Tjandra, P. M. ter Wee, and
F. T. de Charro. "Economic Impact of Extended Time on Peritoneal Dialysis as a Result of Using Polyglucose: The Application of a Markov Chain Model to Forecast Changes in the Development of the Esrd Programme over Time." *Nephrol Dial Transplant* 18, no. 2 (Feb 2003): 390-6.

European Renal Association - European Dialysis and Transplant Association (ERA-EDTA)
http://www.era-edta-reg.org/index.jsp?p=10

"From Era-Edta-Transplantation: Calcification Propensity Associated with Renal Graft Failure." *Nat Rev Nephrol* (Jun 17 2014).

Maggiore, U., R. Oberbauer, J. Pascual, O. Viklicky, C. Dudley, K. Budde, S. S. Sorensen*, et al.* "Strategies to Increase the Donor Pool and Access to Kidney Transplantation: An International Perspective." *Nephrol Dial Transplant* (Jun 6 2014).

Spithoven, E. M., A. Kramer, E. Meijer, B. Orskov, C. Wanner, F. Caskey, F. Collart*, et al.* "Analysis of Data from the Era-Edta Registry Indicates That Conventional Treatments for Chronic Kidney Disease Do Not Reduce the Need for Renal Replacement Therapy in Autosomal Dominant Polycystic Kidney Disease." *Kidney Int* (May 14 2014).

Finnish Registry for Kidney Diseases
http://www.musili.fi/munuais-_ja_maksaliitto/munuaistautirekisteri/finnish_registry_for_kidney_diseases

Helve, J., M. Haapio, P. H. Groop, C. Gronhagen-Riska, and P. Finne. "Comorbidities and Survival of Patients with Type 1 Diabetes on Renal Replacement Therapy." *Diabetologia* 54, no. 7 (Jul 2011): 1663-9.

Helve, J., R. Sund, M. Haapio, P. H. Groop, C. Gronhagen-Riska, and P. Finne. "Medication among Patients with Type 1 Diabetes and Predialytic Renal Disease." *Diabetes Res Clin Pract* 103, no. 3 (Mar 2014): 510-5.

French Renal Epidemiology and Information Network (REIN)
http://www.soc-nephrologie.org/REIN/

Assogba, F. G., C. Couchoud, T. Hannedouche, E. Villar, L. Frimat, A. Fagot-Campagna, C. Jacquelinet, and B. Stengel. "Trends in the Epidemiology and Care of Diabetes Mellitus-Related End-Stage Renal Disease in France, 2007-2011." *Diabetologia* 57, no. 4 (Apr 2014): 718-28.

Mourad, G., J. Minguet, V. Pernin, V. Garrigue, M. N. Peraldi, M. Kessler, C. Jacquelinet*, et al.* "Similar Patient Survival Following Kidney Allograft Failure Compared with Non-Transplanted Patients." *Kidney Int* (Feb 19 2014).

Romeu, M., C. Couchoud, J. C. Delaroziere, S. Burtey, L. Chiche, J. R. Harle, B. Gondouin*, et al.* "Survival of Patients with Anca-Associated Vasculitis on Chronic Dialysis: Data from the French Rein Registry from 2002 to 2011." *QJM* (Mar 19 2014).

Greek Registry (Hellenic Society of Nephrology)
http://www.ene.gr/

Stewart, J. H., M. R. McCredie, S. M. Williams, S. S. Fenton, L. Trpeski, S. P. McDonald, K. J. Jager*, et al.* "The Enigma of Hypertensive Esrd: Observations on Incidence and Trends in 18 European, Canadian, and Asian-Pacific Populations, 1998 to 2002." *Am J Kidney Dis* 48, no. 2 (Aug 2006): 183-91.

Groupement des Nephrologues Francophones de Belgique (GNFB)
http://www.gnfb.be/admin/adminRapportREG.php

Nortier, J., P. Peeters, F. Collart, J. M. Des Grottes, and J. M. Pochet. "[World Kidney Day 2008: The Impact of Epidemiology of Kidney Diseases in Belgium]." *Rev Med Brux* 29, no. 1 Suppl (2008): S19-24.

Italian Dialysis and Transplant Registry (RIDT)
http://www.sin-ridt.org/web/eventi/RIDT/index.cfm

Limido, A. "[Italian Dialysis and Transplant Registry (Ridt): Current Activities and Future Plans]." *G Ital Nefrol* 28, no. 2 (Mar-Apr 2011): 124-5.

Limido, A., M. Nichelatti, M. Postorino, J. H. Levialdi Ghiron, A. Di Napoli, U. Maggiore, M. Nordio, G. Quintaliani, and A. Rustici. "[Changes in the Uremic Population Overtime: Data from the Italian Registry of Dialysis and Transplantation (Ridt)]." *G Ital Nefrol* 29 Suppl 58 (Nov-Dec 2012): S21-6.

Norwegian Renal Registry
http://www.nephro.no/

Knoop, T., B. E. Vikse, E. Svarstad, S. Leh, A. V. Reisaeter, and R. Bjorneklett. "Mortality in Patients with Iga Nephropathy." *Am J Kidney Dis* 62, no. 5 (Nov 2013): 883-90.

Laegreid, I. K., K. Aasarod, A. Bye, T. Leivestad, and M. Jordhoy. "The Impact of Nutritional Status, Physical Function, Comorbidity and Early Versus Late Start in Dialysis on Quality of Life in Older Dialysis Patients." *Ren Fail* 36, no. 1 (Feb 2014): 9-16.

Vikse, B. E., L. M. Irgens, S. A. Karumanchi, R. Thadhani, A. V. Reisaeter, and R. Skjaerven. "Familial Factors in the Association between Preeclampsia and Later Esrd." *Clin J Am Soc Nephrol* 7, no. 11 (Nov 2012): 1819-26.

Peritoneal Dialysis Board Registry (GSDP)
http://www.dialisiperitoneale.org/

Portuguese Society of Nephrology
http://www.spnefro.pt/

Romanian Renal Registry (RRR)
http://srnefro.ro/index.html

Mircescu, G., D. Capsa, M. Covic, M. G. Caprioara, G. Gluhovschi, O. Golea, N. Ursea*, et al.* "Nephrology and Renal Replacement Therapy in Romania--Transition Still Continues (Cinderella Story Revisited)." *Nephrol Dial Transplant* 19, no. 12 (Dec 2004): 2971-80.

Mircescu, G., G. Stefan, L. Garneata, I. Mititiuc, D. Siriopol, and A. Covic. "Outcomes of Dialytic Modalities in a Large Incident Registry Cohort from Eastern Europe: The Romanian Renal Registry." *Int Urol Nephrol* 46, no. 2 (Feb 2014): 443-51.

Russian Registry
http://www.nephro.ru/

Scottish Renal Registry (SRR)
http://www.srr.scot.nhs.uk/

Boyd, J., M. W. Mackinnon, A. Severn, J. P. Traynor, C. E. Whitworth, and W. Metcalfe. "The Delivery of Renal Replacement Therapy in Scotland--Why the Geographic Variation?" *QJM* 106, no. 12 (Dec 2013): 1077-85.

Bray, B. D., J. Boyd, C. Daly, K. Donaldson, A. Doyle, J. G. Fox, A. Innes*, et al.* "Vascular Access Type and Risk of Mortality in a National Prospective Cohort of Haemodialysis Patients." *QJM* 105, no. 11 (Nov 2012): 1097-103.

Bray, B. D., J. Boyd, C. Daly, A. Doyle, K. Donaldson, J. G. Fox, A. Innes*, et al.* "How Safe Is Renal Replacement Therapy? A National Study of Mortality and Adverse Events Contributing to the Death of Renal Replacement Therapy Recipients." *Nephrol Dial Transplant* 29, no. 3 (Mar 2014): 681-7.

Spanish Society of Nephrology Register (Peritoneal Dialysis Registry) (SEN)
http://www.senefro.org/

Garcia de Lorenzo, A., J. Arrieta, A. Ayucar, G. Barril, and E. Huarte. "[Intra-Dialysis Parenteral Nutrition in Chronic Renal Patients: Consensus Sen-Senpe]." *Nutr Hosp* 25, no. 3 (May-Jun 2010): 375-7.

Hervas Sanchez, J. G., M. D. Prados Garrido, A. Polo Moyano, and S. Cerezo Morales. "Effectiveness of Treatment with Oral Paricalcitol in Patients with Pre-Dialysis Chronic Kidney Disease." *Nefrologia* 31, no. 6 (2011): 697-706.

Spanish Society of Nephrology Register (Records of Renal Patients [GRER])
http://www.senefro.org/

Swedish Renal Registry (SNR/SRR)
http://www.medscinet.net/snr/

Aiff, H., P. O. Attman, M. Aurell, H. Bendz, S. Schon, and J. Svedlund. "End-Stage Renal Disease Associated with Prophylactic Lithium Treatment." *Eur Neuropsychopharmacol* 24, no. 4 (Apr 2014): 540-4.

Aiff, H., P. O. Attman, M. Aurell, H. Bendz, S. Schon, and J. Svedlund. "The Impact of Modern Treatment Principles May Have Eliminated Lithium-Induced Renal Failure." *J Psychopharmacol* 28, no. 2 (Feb 2014): 151-4.

Akaberi, S., N. Clyne, G. Sterner, B. Rippe, E. Reihner, P. Wagner, R. Rylance, K. G. Prutz, and M. Almquist. "Temporal Trends and Risk Factors for Parathyroidectomy in the Swedish Dialysis and Transplant Population - a Nationwide, Population-Based Study 1991 - 2009." *BMC Nephrol* 15 (2014): 75.

Turkish National Registry (TSNRR)
http://tsn.org.tr/en/index.php?cat=7

Connor, T. M., D. D. Oygar, D. P. Gale, R. Steenkamp, D. Nitsch, G. H. Neild, and P. H. Maxwell. "Incidence of End-Stage Renal Disease in the Turkish-Cypriot Population of Northern Cyprus: A Population Based Study." *PLoS One* 8, no. 1 (2013): e54394.

Suleymanlar, G., K. Serdengecti, M. R. Altiparmak, K. Jager, N. Seyahi, and E. Erek. "Trends in Renal Replacement Therapy in Turkey, 1996-2008." *Am J Kidney Dis* 57, no. 3 (Mar 2011): 456-65.

United Kingdom Renal Registry (UKRR)
http://www.renalreg.org

Castledine, C. I., J. A. Gilg, C. Rogers, Y. Ben-Shlomo, and F. J. Caskey. "Renal Centre Characteristics and Physician Practice Patterns Associated with Home Dialysis Use." *Nephrol Dial Transplant* 28, no. 8 (Aug 2013): 2169-80.

Nicholas, J., C. Shaw, D. Pitcher, and A. Dawnay. "Uk Renal Registry 16th Annual Report: Chapter 12 Biochemical Variables Amongst Uk Adult Dialysis Patients in 2012: National and Centre-Specific Analyses." *Nephron Clin Pract* 125, no. 1-4 (2013): 219-58.

Rao, A., D. Pitcher, and K. Farrington. "Uk Renal Registry 16th Annual Report: Chapter 11 Blood Pressure Profile of Prevalent Patients Receiving Renal Replacement Therapy in 2012: National and Centre-Specific Analyses." *Nephron Clin Pract* 125, no. 1-4 (2013): 209-18.

Valencian Renal Registry
http://www.sp.san.gva.es/epidemiologia/

Stewart, J. H., M. R. McCredie, S. M. Williams, S. S. Fenton, L. Trpeski, S. P. McDonald, K. J. Jager*, et al.* "The Enigma of Hypertensive Esrd: Observations on Incidence and Trends in 18 European, Canadian, and Asian-Pacific Populations, 1998 to 2002." *Am J Kidney Dis* 48, no. 2 (Aug 2006): 183-91.

LATIN/SOUTH AMERICA

Argentina Registry of Chronic Dialysis
http://www.san.org.ar/

National Information System: National Registry of ESRD (SINTRA)
https://irct.incucai.gov.ar/public/Modulo1.do

Bisigniano, L., A. Lopez-Rivera, V. Tagliafichi, V. Fernandez, and C. Soratti. "Factors Associated with Patient and Graft Survival Using Kidneys from Cadaveric Donors in Transplant Patients under 18 Years of Age in Argentina." *Transplant Proc* 44, no. 7 (Sep 2012): 2235-8.

Chronic kidney disease in Latin America: time to improve screening and detection. *Clin J Am Soc Nephrol* 3, no. 2 (Mar 2008): 594-600.

Marinovich, S., C. Lavorato, G. Rosa-Diez, L. Bisigniano, V. Fernandez, and D. Hansen-Krogh. "The Lack of Income Is Associated with Reduced Survival in Chronic Haemodialysis." *Nefrologia* 32, no. 1 (2012): 79-88.

Marinovich, S., J. Perez-Loredo, C. Lavorato, G. Rosa-Diez, L. Bisigniano, V. Fernandez, and D. Hansen-Krogh. "Initial Glomerular Filtration Rate and Survival in Hemodialysis. The Role of Permanent Vascular Access." *Nefrologia* 34, no. 1 (2014): 76-87.

Latin American Dialysis and Renal Transplant Registry: 2008 report (data 2006). *Clin Nephrol*, no. 74 (Nov 2010): Suppl 1:S3-8.

Latin-American Dialysis and Kidney Transplantation Registry: data on the treatment of end-stage renal disease in Latin America. *G Ital Nefrol* 25, no. 5 (Sept−Oct 2008): 547-53.

Brazilian Registry of Dialysis (RBD/SBN)
http://www.sbn.org.br/

Cordeiro, A. C., J. J. Carrero, A. R. Qureshi, R. F. Cunha, B. Lindholm, Id Castro, and I. L. Noronha. "Study of the Incidence of Dialysis in Sao Paulo, the Largest Brazilian City." *Clinics (Sao Paulo)* 68, no. 6 (Jun 2013): 760-5.

dos Reis Santos, I., A. R. Danaga, I. de Carvalho Aguiar, E. F. Oliveira, I. S. Dias, J. J. Urbano, A. A. Martins*, et al.* "Cardiovascular Risk and Mortality in End-Stage Renal Disease Patients Undergoing Dialysis: Sleep Study, Pulmonary Function, Respiratory Mechanics, Upper Airway Collapsibility, Autonomic Nervous Activity, Depression, Anxiety, Stress and Quality of Life: A Prospective, Double Blind, Randomized Controlled Clinical Trial." *BMC Nephrol* 14 (2013): 215.

Colombia Healthcare Database
http://www.cuentadealtocosto.org/

Latin American Dialysis and Transplantation Registry (SLANH/RLDT)
http://www.slanh.org/index.php/registros

Cusumano, A., G. G. Garcia, C. Di Gioia, O. Hermida, and C. Lavorato. "The Latin American Dialysis and Transplantation Registry (Rldt) Annual Report 2004." *Ethn Dis* 16, no. 2 Suppl 2 (Spring 2006): S2-10-3.

Cusumano, A., G. Garcia Garcia, and C. Gonzalez Bedat. "The Latin American Dialysis and Transplant Registry: Report 2006." *Ethn Dis* 19, no. 1 Suppl 1 (Spring 2009): S1-3-6.

Cusumano, A. M., C. Di Gioia, O. Hermida, and C. Lavorato. "The Latin American Dialysis and Renal Transplantation Registry Annual Report 2002." *Kidney Int Suppl*, no. 97 (Aug 2005): S46-52.

Uruguayan Registry of Dialysis
http://www.nefrouruguay.com/index.php?option=com_content&task=view&id=53&Itemid=277

Gonzalez, C., J. Fernandez-Cean, F. Gonzalez-Martinez, E. Schwedt, and N. Mazzuchi. "[Chronic Dialysis in Uruguay: Mortality Trends from 1981 to 1998]]." *Nefrologia* 21, no. 4 (Jul-Aug 2001): 342-8.

Mazzuchi, N., C. Gonzalez, F. Gonzalez-Martinez, E. Schwedt, A. Correa, F. Correa, and J. Fernandez-Cean. "[Significance of Comorbidity in the Control of the Quality of Treatment of Dialysis Patients]." *Nefrologia* 21, no. 5 (Sep-Oct 2001): 471-5.

MIDDLE EAST

United Arab Emirates Renal Diseases Registry

Yahya, T. M., A. Pingle, Y. Boobes, and S. Pingle. "Analysis of 490 Kidney Biopsies: Data from the United Arab Emirates Renal Diseases Registry." *J Nephrol* 11, no. 3 (May-Jun 1998): 148-50.

NORTH AMERICA

British Columbia Renal Database - Patient Records and Outcome Management (PROMIS)
Information System
http://www.bcrenalagency.ca/professionals/promis/default.htm

Copland, M., D. Murphy-Burke, A. Levin, R. S. Singh, P. Taylor, and L. Er. "Implementing a Home Haemodialysis Programme without Adversely Affecting a Peritoneal Dialysis Programme." *Nephrol Dial Transplant* 24, no. 8 (Aug 2009): 2546-50.

Levin, A., O. Djurdjev, J. Duncan, D. Rosenbaum, and R. Werb. "Haemoglobin at Time of Referral Prior to Dialysis Predicts Survival: An Association of Haemoglobin with Long-Term Outcomes." *Nephrol Dial Transplant* 21, no. 2 (Feb 2006): 370-7.

Canadian Organ Replacement Register (CORR)
http://www.cihi.ca/CIHI-ext-portal/internet/en/document/types+of+care/specialized+services/organ+replacements/corr_metadata

Nessim, S. J., J. M. Bargman, S. V. Jassal, M. J. Oliver, Y. Na, and J. Perl. "The Impact of Transfer from Hemodialysis on Peritoneal Dialysis Technique Survival." *Perit Dial Int* (Dec 1 2013).

Sela, N., K. P. Croome, N. Chandok, P. Marotta, W. Wall, and R. Hernandez-Alejandro. "Changing Donor Characteristics in Liver Transplantation over the Last 10 Years in Canada." *Liver Transpl* 19, no. 11 (Nov 2013): 1236-44.

Zhang, J. C., A. A. Al-Jaishi, Y. Na, E. de Sa, and L. M. Moist. "Association between Vascular Access Type and Patient Mortality among Elderly Patients on Hemodialysis in Canada." *Hemodial Int* (Mar 18 2014).

Canadian Pediatric End-Stage Renal Disease Database

Samuel, S. M., M. A. Tonelli, B. J. Foster, A. Nettel-Aguirre, Y. Na, R. Williams, A. Soo, and B. R. Hemmelgarn. "Overview of the Canadian Pediatric End-Stage Renal Disease Database." *BMC Nephrol* 11 (2010): 21.

Database of the Renal Research Institute (MONDO)
http://renalresearch.com/RRI/index.htm

Usvyat, L. A., P. Kotanko, F. M. van der Sande, J. P. Kooman, M. Carter, K. M. Leunissen, and N. W. Levin. "Circadian Variations in Body Temperature During Dialysis." *Nephrol Dial Transplant* 27, no. 3 (Mar 2012): 1139-44.

North American Pediatric Renal Trials and Collaborative Studies (NAPRTCS)
http://spitfire.emmes.com/study/ped/index.htm

Chen, A., K. Martz, and P. S. Rao. "Does Allograft Failure Impact Infection Risk on Peritoneal Dialysis: A North American Pediatric Renal Trials and Collaborative Studies Study." *Clin J Am Soc Nephrol* 7, no. 1 (Jan 2012): 153-7.

Kim, J. J., and S. D. Marks. "Long-Term Outcomes of Children after Solid Organ Transplantation." *Clinics (Sao Paulo)* 69 Suppl 1 (Jan 2014): 28-38.

Smith, J. M., K. Martz, and T. D. Blydt-Hansen. "Pediatric Kidney Transplant Practice Patterns and Outcome Benchmarks, 1987-2010: A Report of the North American Pediatric Renal Trials and Collaborative Studies." *Pediatr Transplant* 17, no. 2 (Mar 2013): 149-57.

The Renal Disease Registry (TRDR/ORN)
http://www.renalnetwork.on.ca/

McQuillan, R., L. Trpeski, S. Fenton, and C. E. Lok. "Modifiable Risk Factors for Early Mortality on Hemodialysis." *Int J Nephrol* 2012 (2012): 435736.

US Renal Data System (USRDS)
http://www.usrds.org/

Gomez-Puerta, J. A., S. S. Waikar, D. H. Solomon, J. Liu, G. S. Alarcon, W. C. Winkelmayer, and K. H. Costenbader. "Erythropoiesis-Stimulating Agent Use among Patients with Lupus Nephritis Approaching End-Stage Renal Disease." *J Clin Cell Immunol* 4, no. 6 (Dec 1 2013): 179.

Thomas, A., and L. E. Peterson. "Reduction of Costs for Anemia-Management Drugs Associated with the Use of Ferric Citrate." *Int J Nephrol Renovasc Dis* 7 (2014): 191-201.

Yan, G., K. C. Norris, T. Greene, A. J. Yu, J. Z. Ma, W. Yu, and A. K. Cheung. "Race/Ethnicity, Age, and Risk of Hospital Admission and Length of Stay During the First Year of Maintenance Hemodialysis." *Clin J Am Soc Nephrol* (Jun 19 2014).

MULTIREGIONAL

Dialysis Outcomes and Practice Patterns Study (DOPPS)
http://www.dopps.org/annualreport/index.htm

Rayner, H. C., R. L. Pisoni, B. W. Gillespie, D. A. Goodkin, T. Akiba, T. Akizawa, A. Saito, E. W. Young, and F. K. Port. "Creation, Cannulation and Survival of Arteriovenous Fistulae: Data from the Dialysis Outcomes and Practice Patterns Study." *Kidney Int* 63, no. 1 (Jan 2003): 323-30.

Saran, R., D. M. Dykstra, R. A. Wolfe, B. Gillespie, P. J. Held, and E. W. Young. "Association between Vascular Access Failure and the Use of Specific Drugs: The Dialysis Outcomes and Practice Patterns Study (Dopps)." *Am J Kidney Dis* 40, no. 6 (Dec 2002): 1255-63.

International Pediatric Peritoneal Dialysis Network registry (IPPN)
http://www.pedpd.org/

Neu, A. M., A. Sander, D. Borzych-Duzalka, A. R. Watson, P. G. Valles, I. S. Ha, H. Patel*, et al.* "Comorbidities in Chronic Pediatric Peritoneal Dialysis Patients: A Report of the International Pediatric Peritoneal Dialysis Network." *Perit Dial Int* 32, no. 4 (Jul-Aug 2012): 410-8.

Schaefer, F., D. Borzych-Duzalka, M. Azocar, R. L. Munarriz, L. Sever, N. Aksu, L. S. Barbosa*, et al.* "Impact of Global Economic Disparities on Practices and Outcomes of Chronic Peritoneal Dialysis in Children: Insights from the International Pediatric Peritoneal Dialysis Network Registry." *Perit Dial Int* 32, no. 4 (Jul-Aug 2012): 399-409.

International Quotidian Dialysis Registry (IQDR)
http://www.quotidiandialysis.org/

Lindsay, R. M., and G. Nesrallah. "The International Quotidian Dialysis Registry." *Nephrol News Issues* 25, no. 12 (Nov 2011): 19-20.

Nesrallah, G. E., R. M. Lindsay, M. S. Cuerden, A. X. Garg, F. Port, P. C. Austin, L. M. Moist*, et al.* "Intensive Hemodialysis Associates with Improved Survival Compared with Conventional Hemodialysis." *J Am Soc Nephrol* 23, no. 4 (Apr 2012): 696-705.

Suri, R. S., R. M. Lindsay, B. A. Bieber, R. L. Pisoni, A. X. Garg, P. C. Austin, L. M. Moist*, et al.* "A Multinational Cohort Study of in-Center Daily Hemodialysis and Patient Survival." *Kidney Int* 83, no. 2 (Feb 2013): 300-7.

French Language Peritoneal Dialysis Registry (RDPLF)
http://www.rdplf.org/

Bechade, C., L. Guittet, D. Evans, C. Verger, J. P. Ryckelynck, and T. Lobbedez. "Early Failure in Patients Starting Peritoneal Dialysis: A Competing Risks Approach." *Nephrol Dial Transplant* (Sep 26 2013).

Lobbedez, T., C. Verger, J. P. Ryckelynck, E. Fabre, and D. Evans. "Outcome of the Sub-Optimal Dialysis Starter on Peritoneal Dialysis. Report from the French Language Peritoneal Dialysis Registry (Rdplf)." *Nephrol Dial Transplant* 28, no. 5 (May 2013): 1276-83.

Verger, C., M. Duman, P. Y. Durand, G. Veniez, E. Fabre, and J. P. Ryckelynck. "Influence of Autonomy and Type of Home Assistance on the Prevention of Peritonitis in Assisted Automated Peritoneal Dialysis Patients. An Analysis of Data from the French Language Peritoneal Dialysis Registry." *Nephrol Dial Transplant* 22, no. 4 (Apr 2007): 1218-23.
